# HDAC Inhibitors Enhance the Chemosensitivity of Osteosarcoma Cells to Etoposide by Suppressing the Hippo/YAP Signaling Pathway

**DOI:** 10.3390/ijms26188935

**Published:** 2025-09-13

**Authors:** Zhijie Cao, Yulu Chen, Mengshan Chen, Qianjin Fan, Hui Sun, Dong Jin, Yajing Liu, Yanwen Xiong, Donglai Wang

**Affiliations:** 1 National Institute for Communicable Disease Control and Prevention, Chinese Center for Disease Control and Prevention, Beijing 102206, China; caozhijie@icdc.cn (Z.C.); chenyulu86@163.com (Y.C.); 1120210665@mail.nankai.edu.cn (M.C.); fanqianjin95@126.com (Q.F.); sunhui@icdc.cn (H.S.); jindong@icdc.cn (D.J.); 2 Institute of Public Health, School of Medicine, Nankai University, Tianjin 300071, China; 3 State Key Laboratory of Common Mechanism Research for Major Diseases & Department of Medical Genetics, Institute of Basic Medical Sciences & School of Basic Medicine, Chinese Academy of Medical Sciences & Peking Union Medical College, Beijing 100005, China; lyj93@ibms.pumc.edu.cn

**Keywords:** osteosarcoma, chemoresistance, combination therapy, anti-tumor, HDAC inhibitor, etoposide

## Abstract

Osteosarcoma primarily occurs in children and adolescents, and is a highly aggressive bone tumor, particularly presenting challenges in metastatic or recurrent cases due to chemoresistance. Emerging evidences suggest that histone deacetylase inhibitors (HDACis) may exert anti-tumor effects by enhancing the efficacy of various therapeutic modalities. However, the combination of traditional chemotherapy with HDACi-based treatment for osteosarcoma intervention has not been thoroughly explored. This study investigates the anticancer properties of HDACis and/or etoposide (VP16) on the osteosarcoma cell lines U2OS and SJSA-1. Cell viability, morphology, growth and apoptosis were evaluated after treatments, in addition to their influence on the expression levels of proteins associated with apoptotic processes. To elucidate the underlying mechanisms, we employed RNA sequencing, RT-qPCR, and Western blot analyses. Treatment with either HDACis or VP16 alone resulted in an antiproliferative effects in U2OS and SJSA-1 cell lines. Notably, HDACis significantly increased the sensitivity of osteosarcoma cells to VP16, as evidenced by marked differences in cell viability, growth, morphology and apoptosis. Furthermore, when compared to doxorubicin treatment, this VP16/TSA/NAM combinatory regimen demonstrated a comparable ability to suppress cell viability while exhibiting a more pronounced inhibition of cell proliferation. Mechanistically, the combination of HDACis and VP16 specifically resulted in inhibition of the Hippo/YAP signaling cascade, accompanied by a reduction in total YAP1 protein expression. Collectively, our findings suggest that HDACis potentiate the capacity of VP16 to hinder cellular proliferation and trigger apoptosis via the downregulation of the Hippo/YAP pathway, thereby providing a prospective approach to overcome chemoresistance in osteosarcoma.

## 1. Introduction

Osteosarcoma is recognized as the predominant malignant tumor of bone [1]. Despite significant advancements in multimodal treatment strategies that integrate surgical intervention with neoadjuvant and adjuvant chemotherapy, the five-year survival rate for non-metastatic osteosarcoma is only about 70%. In addition, osteosarcoma exhibits a limited responsiveness to the first-line therapeutic regimens, and metastatic or recurrent cases typically have a dismal prognosis due to acquired chemoresistance [1,2,3]. Although novel therapeutic options, including immune checkpoint inhibitors [4], receptor tyrosine kinase inhibitors [5], and highly functionalized xanthenes [6], show promise for the treatment of osteosarcoma, they have not effectively addressed the issue of drug resistance, and their efficacy remains suboptimal. Therefore, it is imperative to identify chemotherapeutic agents with enhanced efficacy.

Recent studies indicate that therapeutic resistance in osteosarcoma is mechanistically linked to epigenetic dysregulation. Epigenetic drugs can serve as novel treatments that enhance the therapeutic efficacy against bone malignancies in clinical settings [7]. The dynamic state of histone acetylation is controlled by the opposing actions of histone acetyltransferases (HATs) and histone deacetylases (HDACs). HDACs, which fall into four classes (I, II, III, and IV), are fundamental regulators of gene expression linked to tumor initiation and advancement. Consequently, they represent critical targets for therapeutic intervention in oncology [8,9].

HDAC inhibitors (HDACis) are particularly effective in inhibiting osteosarcoma cell growth and inducing apoptosis [10,11,12]. Trichostatin A (TSA), an inhibitor of class I and II HDACs, has demonstrated anti-proliferative effects in various malignancies, including osteosarcoma, where it inhibits cell growth and promotes apoptosis [13]. Nicotinamide (NAM), inhibitor of class III HDACs, suppresses proliferation and increases the responsiveness of specific cancer types to anti-tumor agents [14,15]. Notably, studies have indicated that combinations of HDACis can enhance the anti-tumor effects in pancreatic, breast and lung cancers [16,17,18]. HDACis are increasingly valued for their potential to enhance the effectiveness of various therapeutic approaches, such as radiotherapy, chemotherapy, targeted therapy, phototherapy, and immunotherapy [8,19,20].

It is important to note that various chemotherapeutic agents induce apoptosis in osteosarcoma. For instance, etoposide (VP16), a topoisomerase II inhibitor, mediates its anticancer activity by causing DNA strand breaks, thereby influencing apoptosis [21]. Research by Unland et al. has reported that HDACis, such as suberoylanilide hydroxamic acid (SAHA, Vorinostat), significantly enhance the apoptotic effects of VP16 in Ewing sarcomas cells [22]. Moreover, the combination of the HDACi (TSA) and VP16 has been demonstrated to trigger apoptosis in drug-resistant non-small cell lung carcinoma cells [23].

In this study, we aimed to develop effective therapeutic strategies to overcome chemoresistance in osteosarcoma by applying HDACis, specifically TSA and NAM, in combination with VP16. Cell proliferation in the U2OS and SJSA-1 osteosarcoma cell lines was assessed utilizing the CCK-8 assay, cell growth assays, and assessment of morphological alterations. Apoptotic induction by the combined treatment of TSA/NAM and VP16 was substantiated through flow cytometry and Western blot analyses, which demonstrated a significant increase in apoptosis. Additionally, we compared the anti-tumor effects of combination of TSA/NAM and VP16 to doxorubicin. RNA sequencing analysis revealed potential molecular pathways implicated in chemoresistance, notably the Hippo signaling pathway, associated with chemoresistance. Key genes and proteins within the Hippo pathway were further characterized using RT-qPCR and Western blot techniques. Our findings propose and substantiate that HDACis (TSA/NAM) potentiate the chemosensitivity of osteosarcoma cells to VP16 through inhibition of the Hippo signaling pathway.

## 2. Results

### 2.1. HDACis Enhanced the Sensitivity of Osteosarcoma Cells to VP16 to Inhibit Osteosarcoma Cell Proliferation

**Figure 1:**
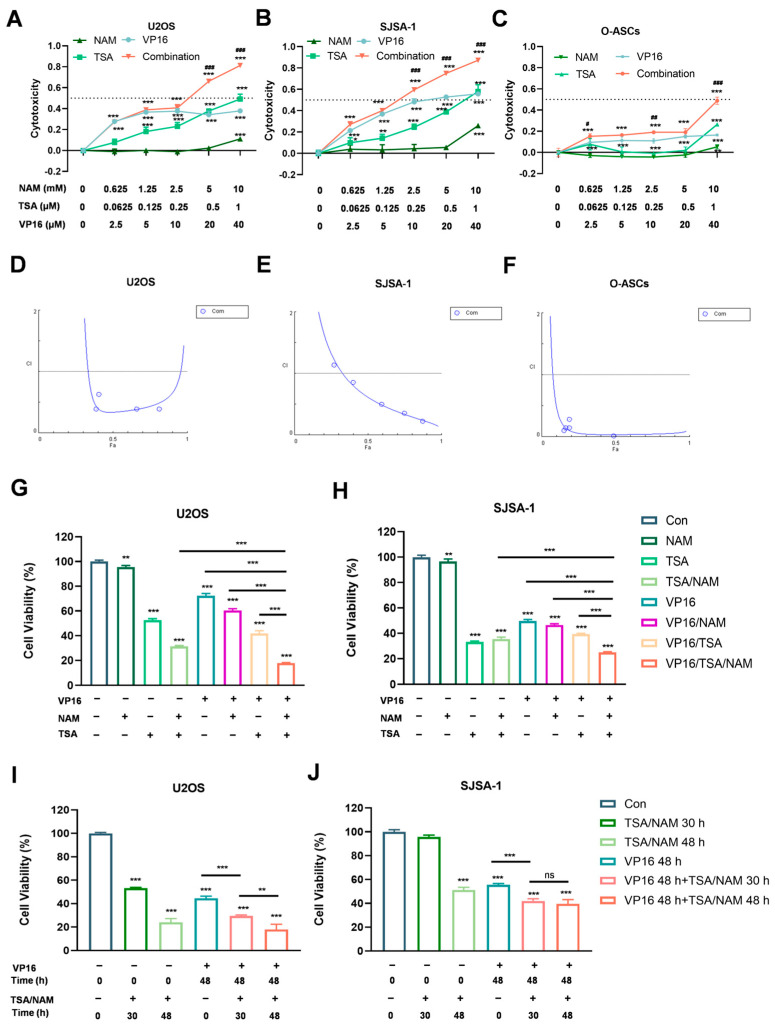
The inhibitory effects of HDACis (TSA and NAM), VP16 and their combination therapy in U2OS and SJSA-1 osteosarcoma cell lines. (**A**–**C**) Cytotoxicity of U2OS, SJSA-1 and O-ASCs cells following treatment with various concentrations of TSA (0.0625, 0.125, 0.25, 0.5, and 1 µM), NAM (0.625, 1.25, 2.5, 5, and 10 mM), and/or VP16 (2.5, 5, 10, 20, and 40 µM) for 72 h. The dotted lines represented a half inhibitory effect. (**D**–**F**) The fraction affected (Fa)-combination index (CI) plots illustrated that HDACis show a clear synergistic interaction with VP16 in U2OS, SJSA-1 and O-ASCs cells for 72 h of co-treatment, as evidenced by the majority of CI values being less than 1. Com, indicates VP16/TSA/NAM combination. (**G**,**H**) Viability of U2OS and SJSA-1 cells after exposure to TSA (1 μM) alone, NAM (5 mM) alone, VP16 (40 μM) alone, and their dual or three drug combinations for 72h. (**I**,**J**) Viability of U2OS and SJSA-1 cells subjected to VP16 treatment for 48 h, and accompany with sequential exposure to TSA/NAM for 30 and 48 h. Data are presented as the mean ± SD from three independent experiments. Statistical significance was calculated using one-way ANOVA followed by Tukey’s multiple comparisons test (ns: not significant, * *p* < 0.05, ** *p* < 0.01, and *** *p* < 0.001). The symbol “#” denotes comparisons between any individual drug and the combination (Com) group, with ## *p* < 0.01, ### *p* < 0.001.

To investigate whether HDACis can enhance the suppressive impact of VP16 on proliferation of osteosarcoma cells, we initially assessed the cell cytotoxicity after HDACis or VP16 alone and their combination. Varying concentrations of TSA, NAM and/or VP16 treated the cells over 72 h, utilizing CCK8 assays to detect. The findings indicated that VP16, TSA or NAM each decreased the cytotoxicity of U2OS and SJSA-1 cells, and in a concentration-dependent manner. Notably, combining HDACis with VP16 yielded greater efficacy than either agent alone (Figure 1A,B). Furthermore, human omental adipose-derived stromal cells (O-ASCs), which share characteristics with mesenchymal stem cells, were served as non-tumoral cells to evaluate the cytotoxic effects of the drugs. The result indicated that the cytotoxicity of O-ASCs was not obviously increased in response to either individual treatment or the combination of three drugs (Figure 1C), compared to the U2OS and SJSA-1 cells (Figure 1A,B).

Synergistic, additive, or antagonistic within the combination treatment were assessed using CompuSyn (Version 1.0, 64-bit) software to calculate the combination index (CI). A CI value below 1 indicates synergy, equal to 1 indicates an additive effect, and above 1 indicates antagonism. The results revealed that CI values predominantly below 1 (Table 1), suggesting that the combination of HDACis, specifically TSA and NAM, with VP16 exerted a synergistic inhibitory effect on the proliferation of U2OS, SJSA-1 cells and O-ASCs under 72 h of exposure (Figure 1D,E). This finding suggests that the osteosarcoma cells, but not the normal cells, preferentially respond to these drugs for cytotoxicity.

Additionally, to determine whether the inhibitory effect of three drugs combination superior to dual drug combinations, we assessed the effects of the three or dual drugs combinations on cell viability. The results demonstrated that either the combinations of VP16/NAM or VP16/TSA significantly decreased cell viability in the U2OS and SJSA-1 cells after 72 h of treatment, in comparison to either control group or VP16 group. Notably, the combination of VP16/TSA/NAM exhibited a markedly greater inhibitory effect on cell viability than the dual drug combinations (Figure 1H,I). These findings suggest that the combination of HDACis enhances the inhibitory effect of VP16 on osteosarcoma cell viability. Furthermore, rather than administering HDACis and VP16 simultaneously, we conducted sequential treatments of HDACis followed by VP16. When U2OS cells were treated with TSA/NAM followed by VP16 after an 18 h delay, a lower inhibition of cell viability was observed compared to simultaneous exposure. However, this delayed treatment did not yield a significant difference in SJSA-1 cells (Figure 1I,J).

### 2.2. HDACis Promoted the Inhibitory of VP16 on the Cell Growth of Osteosarcoma Cells

**Figure 2:**
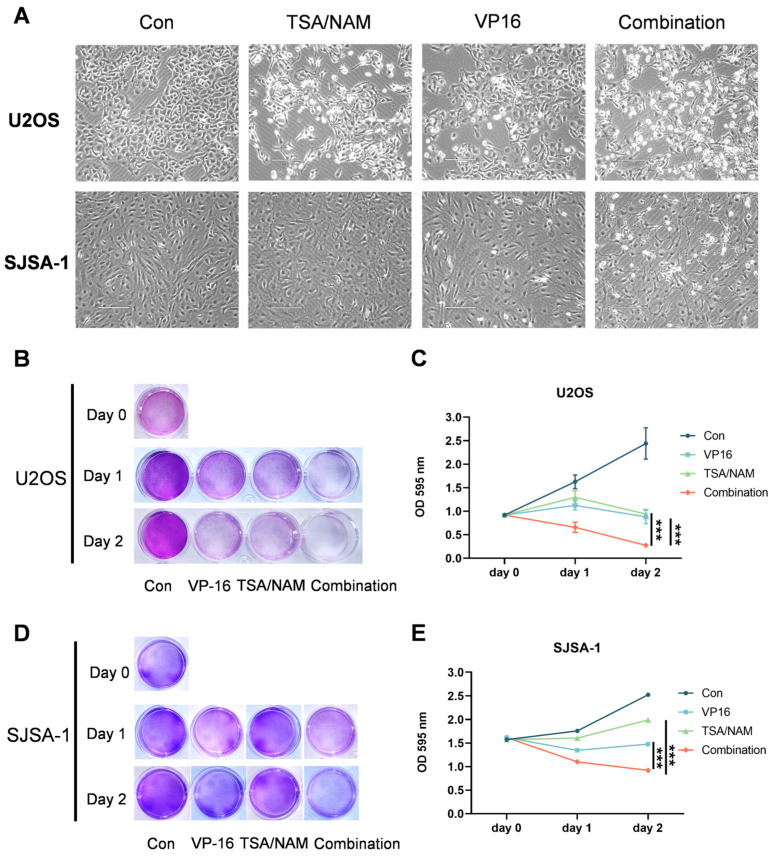
HDACis potentiated the anti-tumor efficacy of VP16 in osteosarcoma cell lines. (**A**) The cell morphology of U2OS and SJSA-1 osteosarcoma cells following 24 h of exposure to HDACis (TSA 1 μM and NAM 5 mM) and/or VP16 (40 μM) using microscopy. Scale bar: 170 μm. (**B**–**E**) The crystal violet staining images alongside quantitative outcomes from cell growth assays conducted on U2OS and SJSA-1 cells. The combination of HDACis (TSA 1 μM and NAM 5 mM) with VP16 (40 μM) effectively inhibit the cell proliferation. All data are presented as mean ± SD from three replicates. Statistical significance was determined using two-way ANOVA followed by Sidak’s multiple comparisons test, and indicated by *** *p* < 0.001.

To directly and intuitively observe the cytotoxic impact of the drugs on osteosarcoma cells, we captured the morphology of U2OS and SJSA-1 cells using microscopy. Individual treatments with TSA/NAM or VP16 for 24 h resulted in a moderate reduction in cellular adhesion, accompanied by partial detachment in U2OS cells, while SJSA-1 cells exhibited minimal morphological alterations. Notably, the combination of these drugs resulted in pronounced cytotoxicity, resulting in extensive cell death in U2OS cells, whereas SJSA-1 cells displayed considerable detachment under combinatorial treatment (Figure 2A). Additionally, crystal violet staining was performed to detect the impact of the drugs on cell growth. Our observations indicated that the combination of TSA/NAM and VP16 markedly impeded the growth of U2OS and SJSA-1 cells, compared to individual treatments with TSA/NAM or VP16 (Figure 2B–E). Consequently, these results demonstrate that HDACis significantly enhance the tumor inhibitory effect of VP16 on cell proliferation.

### 2.3. HDACis Enhanced the Chemosensitivity of VP16 by Inducing Apoptosis of Osteosarcoma Cells

**Figure 3:**
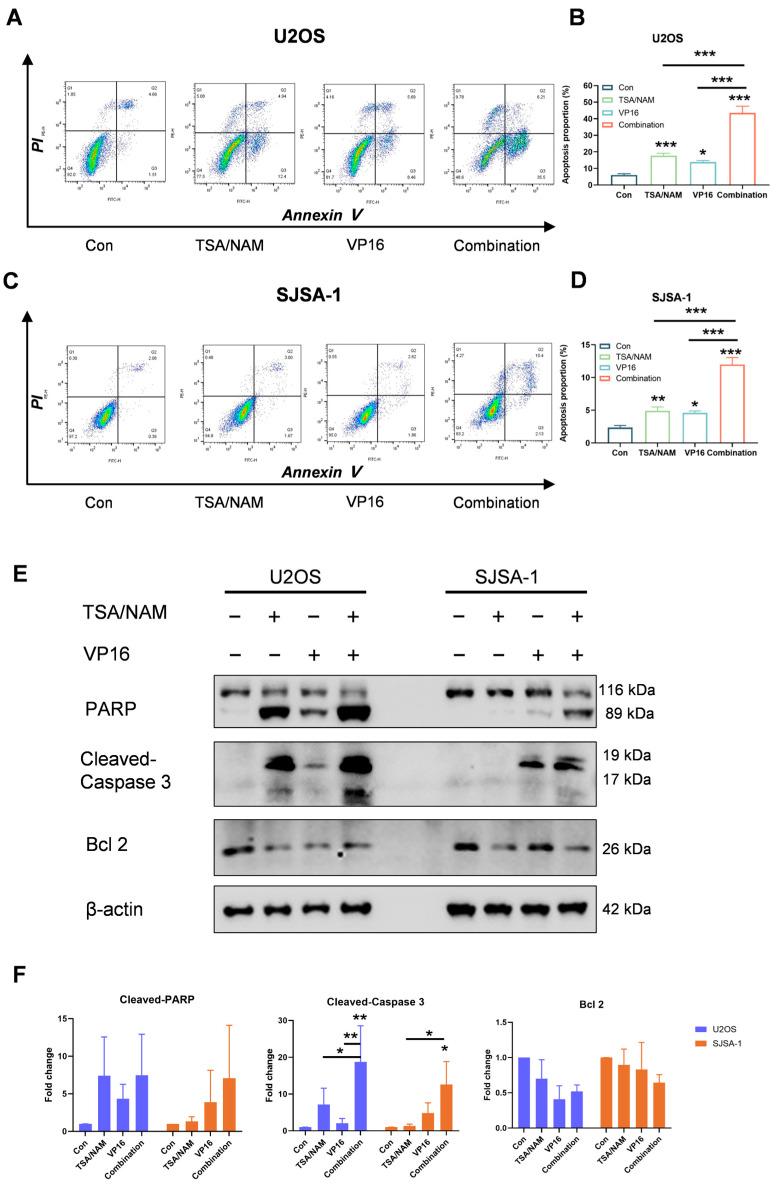
Effect of HDACis and VP16 alone or in combination on apoptosis of osteosarcoma cells. (**A**,**C**) A representative images showed apoptosis in U2OS and SJSA-1 cells after 24 h of treatment with HDACis (TSA 1 μM and NAM 5 mM) and/or VP16 (40 μM). The color scale ranges from blue (low density) to red (high density), indicating the local density of cells. (**B**,**D**) The apoptosis proportion of U2OS and SJSA-1 cells based on (**A**,**C**). (**E**) Western blot assay determined the expression changes in apoptosis related protein including PARP, cleaved-caspase 3, Bcl-2, with beta-actin serving as a reference protein. (**F**) Quantification analysis of the Western blot band intensities, derived from the data in (**E**), showing the relative expression of cleaved-PARP, cleaved-Caspase 3, and Bcl 2 proteins normalized to beta-actin, based on the data presented in (**E**). All data are shown as mean ± SD and are derived from three independent replicates. *P* values were calculated using one-way ANOVA followed by Tukey’s multiple comparisons test, and indicated by * *p* < 0.05, ** *p* < 0.01, *** *p* < 0.001.

To investigate the impact of this combination on apoptosis, cells treated with TSA/NAM and/or VP16 for 24 h were analyzed using Annexin V/PI staining. The results demonstrated that treatment with TSA/NAM markedly elevated the percentage of apoptotic cells in U2OS and SJSA-1 cells. Importantly, the VP16/TSA/NAM combination group showed a significantly greater rate of apoptosis relative to the group treated with VP16 alone, highlighting the enhancing impact of TSA/NAM on apoptosis induction (Figure 3A–D). Furthermore, the expression levels of apoptosis-related markers were analyzed by Western blotting. The results demonstrated a marked increase in cleaved-caspase-3 levels in combination group, while cleaved-PARP levels exhibited an increasing trend in both cell lines. Moreover, U2OS and SJSA-1 cells exposure to the combined treatment exhibited a downward trend in the expression levels of anti-apoptotic protein Bcl-2 (Figure 3E,F). In conclusion, the combination of HDACis and VP16 significantly enhances apoptosis in osteosarcoma cells, underscoring the increased apoptotic induction potential of VP16 when used in conjunction with TSA/NAM.

### 2.4. Combination of HDACis and VP16 Exerted Comparable Inhibitory Effect with Doxorubicin

**Figure 4:**
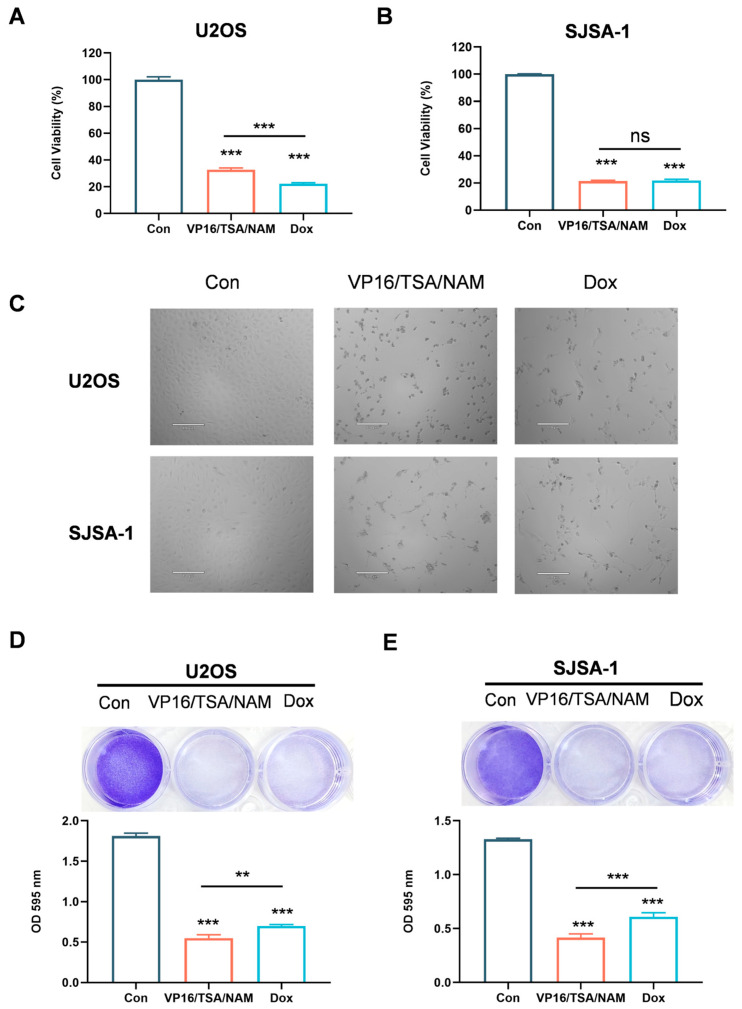
Comparison of the antitumor efficacy of the VP16/TSA/NAM combination therapy versus doxorubicin. (**A**,**B**) The cell viability of U2OS and SJSA-1 cells were assessed for 48 h exposure to combination treatment (TSA 1 μM, NAM 5 mM and VP16 40 μM) or Dox (2 μM). (**C**) The cell morphology of U2OS and SJSA-1 cells after 48 h of exposure to either the combination treatment (TSA 1 μM, NAM 5 mM and VP16 40 μM) or Dox (2 μM), as observed under a microscope. Scale bar: 170 μm. (**D**,**E**) Cell proliferation analysis of U2OS and SJSA-1 cells treated with the combination treatment (TSA 1 μM, NAM 5 mM and VP16 40 μM) or Dox (2 μM) after 48 h. Data are shown as means ± SD with three replicates. ns: not significant. Statistical significance was calculated using one-way ANOVA followed by Tukey’s multiple comparisons test. ** *p* < 0.01, *** *p* < 0.001 indicated the statistics significance.

To compare the therapeutic efficacy of combining HDACis with VP16 against standard chemotherapy in the treatment of osteosarcoma, we conducted CCK-8 assays to measure the viability of U2OS and SJSA-1 cells following exposure to the combination treatment (VP16/TSA/NAM) and a first-line chemotherapeutic agent doxorubicin (Dox). The findings proved both the VP16/TSA/NAM combination and Dox treatments observably suppressed cell proliferation of U2OS and SJSA-1 cells after 48 h exposure (Figure 4A,B). Notably, no significant difference was detected between the VP16/TSA/NAM combination group and Dox group in SJSA-1 cells. Meanwhile, Dox treatment exhibited a marginally greater inhibitory effect compared to the VP16/TSA/NAM combination. Interestingly, alterations in cell morphology were more pronounced following treatment with the VP16/TSA/NAM combination treatment than with Dox treatment (Figure 4C). Moreover, relative to the control group, increased cell death was more pronounced in both the combination and Dox groups, with the combination treatment inducing a marginally higher level of cell death than the Dox group (Figure 4D,E). Altogether, these results demonstrate that the combination of HDACis with VP16 exerts an inhibitory effect on osteosarcoma cells comparable to those of Dox treatment.

### 2.5. Hippo/YAP1 Pathway Involved in HDACi Augmented Sensitivity of Osteosarcoma Cells to VP16

**Figure 5:**
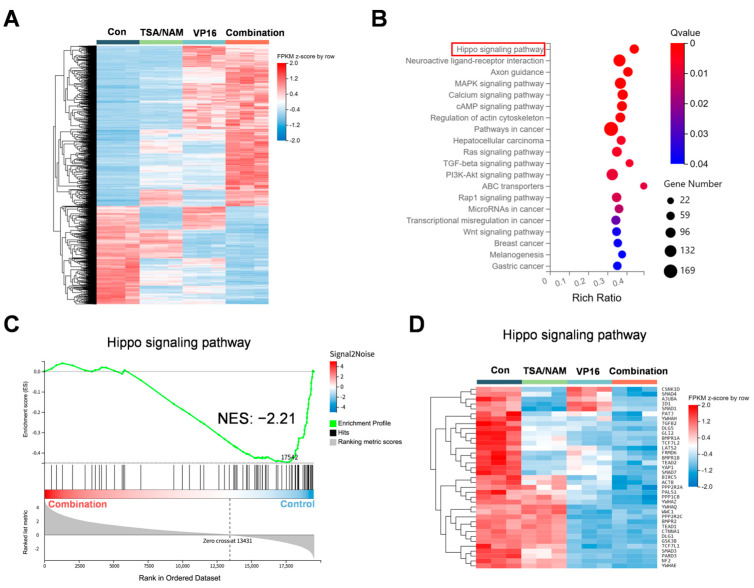
Differentially expressed genes and pathways after combination treatment with HDACis and VP16 in U2OS osteosarcoma cells. (**A**) Heatmap presentation of 4611 differentially expressed genes influenced by the treatments in U2OS cells. (**B**) KEGG analysis pertaining to the differentially expressed genes. Red box highlighted the Top 1 significantly enriched pathway. (**C**) GSEA focusing on the Hippo signaling pathway in cells subjected to combination treatment compared to control cells. (**D**) Differential gene clustering heatmap representing the Hippo signaling pathway in U2OS cells subjected to treatment with HDACis and/or VP16.

To elucidate the mechanism underlying this augmented sensitivity of etoposide, total RNA extracted from U2OS cells was analyzed via RNA sequencing. Differentially expressed genes (DEGs) were screened and visualized using a heatmap (Figure 5A). Subsequent KEGG pathway enrichment analysis highlighted the top 20 enriched pathways among DEGs in combination treatment group relative to the control group. Notably, the Hippo signaling pathway emerged as the top 1 significantly enriched (Figure 5B). Additionally, Gene Set Enrichment Analysis (GSEA) along with the corresponding DEG clustering heatmap, indicated a significant downregulation of Hippo pathway activity following combination treatment (Figure 5C,D).

**Figure 6:**
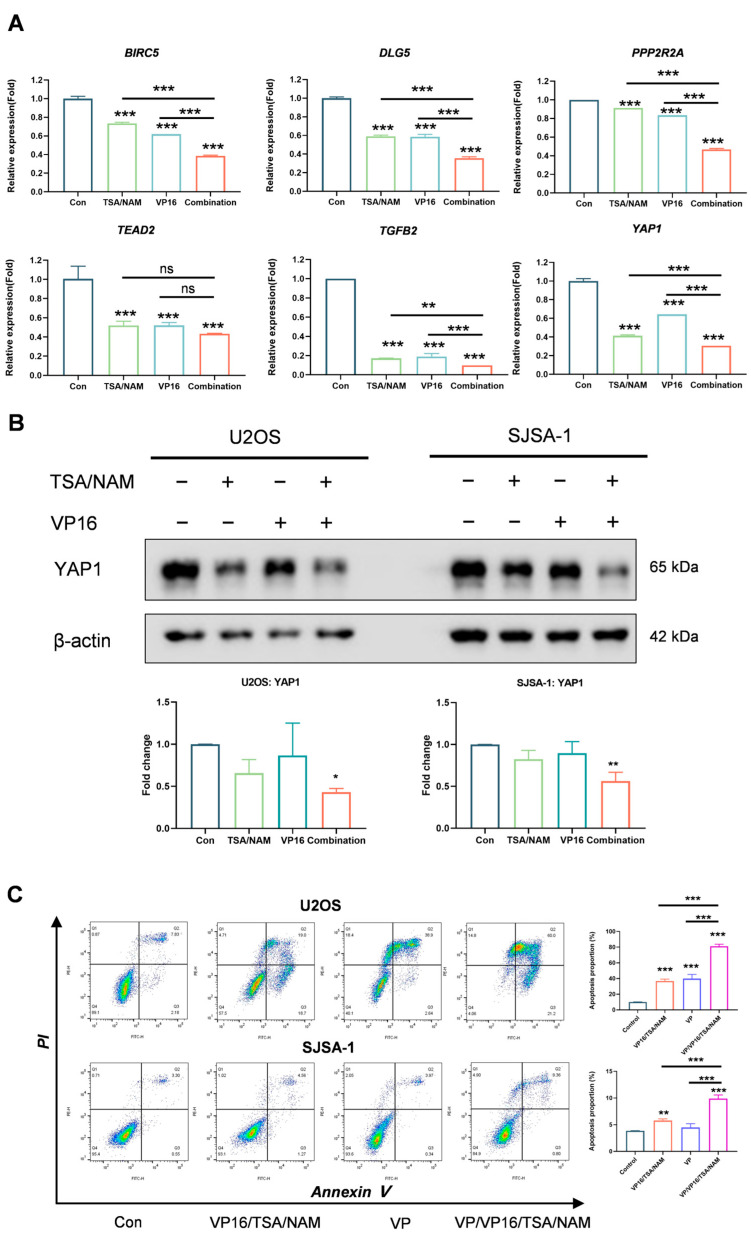
HDACis augmented the sensitivity of osteosarcoma cell to VP16 by suppressing the Hippo pathway. (**A**) RT-qPCR results of U2OS cells treated with HDACis (TSA 1 μM and NAM 5 mM) and/or VP-16 (40 μM) after 24 h of treatments. (**B**) Western blotting and quantification representative protein expression of YAP1 in U2OS and SJSA-1 cells subjected to HDACis and/or VP-16 treatments for 24 h, with beta-actin serving as a reference protein. (**C**) Flowcytometry analysis of osteosarcoma cells after treatment with VP16/TSA/NAM and/or YAP inhibitor (Verteporfin 1 μM) for 24 h. VP, Verteporfin. The color scale, ranging from blue to red, represents the local density of cells, with blue indicating low density and red indicating high density. All data are presented as mean ± SD, with three replicates. Statistical significance was determined using one-way ANOVA followed by Tukey’s multiple comparisons test. ns: not significant. * *p* < 0.05, ** *p* < 0.01, *** *p* < 0.001 indicated the statistics significance.

Subsequently, RT-qPCR was conducted to corroborate the findings derived from the RNA sequencing analysis. After the genes examined, *BIRC5*, *DLG5*, *PPP2R2A*, *TEAD2*, *TGFB2* and *YAP1* exhibited expression patterns consistent with the RNA-seq findings (Figure 6A). Additionally, to determine whether the specific inhibition of Hippo signaling pathway is exclusive to the VP16/TSA/NAM combination, we performed RT-qPCR analysis to detect the expression levels of Hippo pathway-related genes in U2OS and SJSA-1 cell lines following treatment with Dox alone and the Dox/TSA/NAM combination. The findings indicated that, there were no significant differences in gene expression between the two treatment groups in either cell line, except for expression of *YAP1* in U2OS cells (Figure S1A,B). These findings suggest that the Hippo pathway represents a specific mechanism underlying the enhanced response observed with the VP16/TSA/NAM combination.

A comprehensive review of relevant literature revealed that YAP1 may be associated with drug resistance. Consequently, YAP1 was selected as the primary candidate for further investigation, leading to the execution of Western blotting. The Western blot analysis validated the data of RNA sequencing, revealing a marked reduction in YAP1 expression in U2OS and SJSA-1 cells subsequent to the combination treatment (Figure 6B), when in comparison to control group. Importantly, verteporfin, a YAP inhibitor, when combined with VP16/TSA/NAM, augmented the therapeutic efficacy by elevating the proportion of apoptotic cells (Figure 6C). Moreover, verteporfin had no inhibitory effect on combined with neither Dox-treated nor Dox/TSA/NAM combination treated cells (Figure S1C,D). Altogether, our findings reveal that the Hippo/YAP1 signaling pathway is integral to the mechanism by which HDACis specifically potentiate the chemosensitivity of osteosarcoma cells to VP16.

## 3. Discussion

Osteosarcoma represents the most widespread form of bone malignancy [1]. However, emerging evidences indicate that patients with metastatic, recurrent, and refractory osteosarcoma face significant challenges in achieving successful treatment outcomes due to chemoresistance. The implementation of combined therapeutic regimens may offer advantages, particularly for patients who exhibit poor responses to standard treatments. Several studies have advocated the notion that the combination of HDACis can enhance antiproliferative responses and promote apoptosis in various cancer cell types [15,16]. Research has indicated that HDACis increase the susceptibility of Ewing sarcoma cell lines to primary chemotherapeutic agents utilized in antitumor treatment [24]. Furthermore, the combination of anti-tumor drugs and HDACis exerted a synergistic effect of anti-tumor [19,25]. Consequently, we repurposed TSA and NAM, both well-known HDACis, as adjunctive therapeutic agents in conjunction with VP16 to develop a neoadjuvant treatment strategy. The results demonstrate that the combined treatment of TSA/NAM with VP16 exhibits synergistic effects when compared to VP16 monotherapy. However, the mechanisms that contribute to the increased chemosensitivity of VP16 in conjunction with HDACis (TSA/NAM) in osteosarcoma cells are multifaceted and warrant further exploration. Utilizing RNA sequencing, PCR, Western blotting and flow cytometry, this study has demonstrated that HDACis (TSA/NAM) enhance the chemosensitivity of osteosarcoma cell lines to VP16 via suppressing the Hippo/YAP signaling pathway.

TSA has been documented to exert substantial antitumor effects through promoting apoptosis in MG63 osteosarcoma cells [13]. Similarly, treatment with TSA has been reported to decrease cell viability and induce apoptosis in the HOS osteosarcoma cell line [11]. Previous investigations have revealed that NAM can trigger apoptosis in MCF-7 tumor cells [26], and inhibit proliferation while inducing apoptosis, and cause cell cycle arrest in pancreatic cancer cells [15]. Nevertheless, the function of NAM in osteosarcoma has yet to be elucidated. Our findings revealed that NAM had a mild cytotoxicity in osteosarcoma cells after 72 h treatments. Nevertheless, its inhibitory effect was comparatively lower than that of TSA. Previous research has demonstrated that combination treatment of TSA and NAM in lung cancer cells can suppress cell growth and enhance apoptotic cell death [18]. Despite these findings, the functions of the TSA/NAM combination on osteosarcoma cells have not been thoroughly investigated.

Our findings indicate that the TSA/NAM combination significantly enhanced cell cytotoxicity in U2OS osteosarcoma cells, compared to either TSA or NAM treatment alone. However, the TSA/NAM combination did not exhibit a significant difference in cell cytotoxicity when compared to either TSA or NAM treatment alone in SJSA-1 osteosarcoma cell lines. VP16 is a conventional chemotherapy drug recommended for osteosarcoma treatment. Importantly, we demonstrated that either TSA or NAM enhanced the inhibitory effect on cell viability to VP16 in U2OS and SJSA-1 cells for 72 h treatments. Notably, the TSA/NAM combination markedly intensified the inhibitory impact on cell viability in response to VP16 in these cell lines, regardless of comparisons to single agents or the two-drug combinations. Similarly, Unland et al. reported that HDACi (SAHA) synergistically enhanced the antiproliferative effects of VP-16 in Ewing sarcoma cell lines [22]. Smith et al. revealed that HDACi (Panobinostat) exhibits a synergistic effect with chemotherapeutic agents such as VP16 or Doxorubicin, thereby substantially enhancing the therapeutic efficacy of conventional chemotherapy regimens in Ewing sarcoma cells [27]. Moreover, to evaluate the potential synergistic effects of combination therapy, we performed CCK8 assays to analyze the impact of HDACis or VP16 administered individually, as well as in combination on U2OS and SJSA-1 osteosarcoma cell lines. After 72 h treatments, synergistic effect of HDACis and VP16 combination was determined through an analysis of the CI plot. Notably, the combination of these drugs demonstrated selective suppression on osteosarcoma cell line U2OS, as well as chemoresistant osteosarcoma cell line SJSA-1, in contrast to non-tumoral O-ASCs. Additionally, the inhibition of cell viability following TSA/NAM simultaneous treatment was more pronounced than after an 18 h delay treatment.

Crystal violet staining further confirmed that the HDACis which contain TSA and NAM, increased the inhibition of cell growth to VP16 in both U2OS and SJSA-1 cell lines. Moreover, the inhibitory effect of the combination treatment was more prominent in U2OS cells than in SJSA-1 cells after 24 h treatments, as evidenced by cell growth images and morphology. This may be attributed to the fact that SJSA-1 is a chemoresistant osteosarcoma cell line [28]. However, the cell viability inhibitory effects of the combination on U2OS and SJSA-1 are comparable after 72 h treatments. The findings indicate that extended-duration combination therapies may represent a viable treatment strategy for chemoresistant osteosarcoma. Additionally, we demonstrated that combination of TSA/NAM with VP16 exerted comparable effects of cell viability to doxorubicin, with a more pronounced inhibition of cell proliferation.

Previous research exposed that combination of HDACi (TSA) and VP-16 effectively induces apoptosis in lung cancer cells [23]. Apoptosis, recognized as a form of programmed cell death, which is a fundamental mechanism exploited in cancer therapies to promote the elimination of malignant cells [29]. Cancer cells frequently circumvent apoptosis, a characteristic associated with tumor development and resistance to chemotherapy. Our findings demonstrated that the combined treatment of TSA/NAM and VP-16 significantly enhanced proportion of apoptotic cells compared to VP-16 treatment alone in U2OS and SJSA-1 cell lines, as determined by flow cytometry analysis. Anti-apoptotic proteins, including Bcl-2, are frequently found to be overexpressed in cancer cells [30]. In this study, we found that treatments with TSA/NAM and/or VP16 diminished the expression of Bcl-2. Moreover, PARP, a nuclear enzyme involved in DNA repair, when cleaved, serves as a valuable marker for apoptosis, indicating the inactivation of its DNA repair function [31]. Cleaved caspase 3 is critically involved in the process of apoptosis; however, it also performs non-apoptotic functions, such as regulating angiogenesis and influencing chemotherapy resistance in cancer cells [32]. Our results indicated that a significant elevation in cleaved caspase-3 levels was observed, along with a tendency toward increased cleaved PARP, subsequent to the combination treatment. These findings suggested that the combination of TSA/NAM and VP-16 enhances cellular sensitivity to apoptosis induced by VP-16.

To elucidate the mechanism underlying the reduction in chemoresistance through combination administration, RNA sequencing was performed to identify differentially expressed genes. GSEA of the RNA sequencing revealed a prominent suppression of Hippo signaling pathway in comparison to control group. The Hippo signaling pathway serves as a pivotal regulator of cellular proliferation and survival, significantly influencing tumor advancement and the emergence of resistance to therapeutic drugs [33]. The Hippo signaling network contains a serial core kinase comprising mammalian Ste20-like kinases 1/2 (MST1/2) as well as large tumor suppressor kinases 1 and 2 (LATS1/2). Activation of this cascade block the nuclear translocation of yes-associated protein 1 (YAP1) and its paralog transcriptional coactivator with a PDZ-binding motif (TAZ), thereby preventing their interaction with TEAD transcription factors [34,35]. However, pathological overactivation of YAP1/TAZ resulting from Hippo dysregulation is a hallmark of many cancers, promoting tumor initiation, invasive behavior, metastasis, and resistance to therapeutics [34,36]. Furthermore, the Hippo signaling pathway contributes to osteosarcoma chemoresistance, with YAP1 as a promising therapeutic target to overcome this resistance [37].

Xia et al. reported that silencing the YAP/TEAD co-activators enhanced sensitivity of ovarian cancer cells to chemotherapeutic agents, including cisplatin, paclitaxel, and bleomycin [38]. Similarly, knockdown of YAP1 increased susceptibility of esophageal cancer cells to 5-fluorouracil and docetaxel [39]. Furthermore, recent investigations have implicated the Hippo/YAP signaling pathway in mediating chemoresistance in osteosarcoma [37,40,41]. In this study, the heatmap analysis revealed significant downregulation of genes such as *BIRC5*, *DLG5*, *PPP2R2A*, *TEAD2*, *TGFB2* and *YAP1* following VP16/TSA/NAM combination treatment. Additionally, we implemented RT-qPCR to validate the RNA-seq analyses and obtained consistent results. However, almost the gene expressions had no significant differences between Dox and Dox/TSA/NAM combination treatments in U2OS and SJSA-1 cells, except for *YAP1* in U2OS cells. The results imply that the VP16/TSA/NAM combination specifically inhibited the Hippo signaling pathway in osteosarcoma. In addition, Western blot experiments demonstrated a marked reduction in YAP1 expression in the osteosarcoma cells after combination treatment. Morice et al. suggested that YAP inhibitors could constitute a promising therapeutic approach for suppressing osteosarcoma tumor progression [42]. Verteporfin, a YAP inhibitor, was utilized to validate that YAP1 mediates the pro-apoptotic effect of the VP16/TSA/NAM combination treatment. The results exhibited that the combination of verteporfin with VP16/TSA/NAM enhanced the therapeutic effect by increasing the proportion of cell apoptosis. Nevertheless, the combination of verteporfin with either Dox or Dox/TSA/NAM had no significant difference in cell proliferation with Dox or Dox/TSA/NAM combination treatments. As mentioned above, our results demonstrated that the HDACis sensitize osteosarcoma cells to VP16 by downregulating the Hippo/YAP1 signaling pathway.

The findings of this study demonstrate that combination treatment effectively inhibits cell proliferation, as well as promotes apoptosis in osteosarcoma cell line U2OS, as well as in chemoresistant osteosarcoma cell line SJSA-1. However, we acknowledge the absence of patient-derived chemoresistant osteosarcoma cells or primary osteoblasts further to strengthen the biological relevance of our study. To facilitate clinical translation, future studies will validate these findings in patient-derived chemoresistant osteosarcoma models as well as patient-derived organoid models. Additionally, in vivo studies will be conducted to ascertain whether combination therapy should be administered concurrently or sequentially, alongside efforts to identify biomarkers for the stratification of patients. These measures are essential for tailoring therapeutic interventions according to reactions of individual patients.

## 4. Materials and Methods

### 4.1. Cell Culture

The osteosarcoma cell lines U2OS and SJSA1 were obtained from American Type Culture Collection (ATCC). These cell lines were maintained in Dulbecco’s Modified Eagle’s Medium (DMEM, C11995500BT, Gibco, Grand Island, NY, USA) supplemented with 10% fetal bovine serum (FBS, C04001-050, Vivacell, Shanghai, China), penicillin (100 U/mL), and streptomycin (100 µg/mL). The O-ASCs were kindly provided by Peking University People’s Hospital and subsequently cultured in Minimum Essential Medium (α-MEM, C3060-0500, Vivacell) supplemented with 10% FBS and 1% penicillin-streptomycin, as previously described [43]. The cells were maintained at 37 °C in a humidified incubator with an atmosphere containing 5% CO_2_ and were routinely subcultured using 0.25% trypsin-EDTA (25200056, Gibco, Grand Island, NY, USA) when they reached 80–90% confluence. The cells were passaged 2–3 times every week.

### 4.2. CCK-8 Assay

5000 osteosarcoma cells or O-ASCs were seeded into each well of 96-well plates, with a volume of 100 µL per well, and subsequently incubated overnight. Next day, the cells were exposed to different varying concentrations of TSA (T6270, TargetMol, Boston, MA, USA), NAM (72340, Sigma-Aldrich, St. Louis, MO, USA) and/or VP16 (T0132, TargetMol, Boston, MA, USA) for 72 h. Meanwhile, the doses were selected based on previously published studies that demonstrated efficacy on similar cell types [15,18,44]. Afterwards, 10 μL of CCK-8 solution (CK04, Dojindo Laboratories, Tokyo, Japan) was added, then incubation at 37 °C for 1–2 h. Soon afterwards, the optical density was measured at wavelength of 450 nm utilizing a microplate reader (Bio-Tek, Winooski, VT, USA). Cell cytotoxicity was quantified according to the following formula: Cytotoxicity = 1 − (experimental group-blank control)/(control group-blank control).

Moreover, to evaluate the synergistic effect of the VP16 combination with TSA/NAM, the inhibitory effect was determined using the formula: inhibitory effect = 1 − (experimental group-blank control)/(control group-blank control). Subsequently, the effect and drug dose concentration data were imported into CompuSyn 1.0, a software application designed for the analysis of the therapeutic effects of drug combination therapies. The software then generated the CI plot and corresponding CI values.

### 4.3. Cell Growth Assay

To assess cell growth, 1.0–1.5 × 10^5^ U2OS or SJSA-1 cell lines were plated in 12-well plates and subsequently treated with VP16 (40 μM) and/or HDACis (TSA 1 μM and NAM 5 mM) for durations of 0, 24 and 48 h. The cell morphologies were observed and captured images under a fluorescence microscope (ECHO Revolve, San Diego, CA, USA) at 24 h. Cell growth was monitored on days 0, 1 and 2 using crystal violet staining. Specifically, cells were fixed for 20 min with 4% paraformaldehyde on a shaker, then staining with 0.1% crystal violet at room temperature (25 °C) for duration of 30 min. Subsequently, cells were rinsed three times with double-distilled H_2_O, air-dried, and photographed. To make a quantitative analysis, the cells were dissolved in 10% acetic acid while agitated on a shaker for a duration of 30 min. Subsequently, the absorbance of the extracted crystal violet was measured at a wavelength of 595 nm.

### 4.4. Apoptosis Assay

U2OS or SJSA-1 cells (1.5 × 10^5^ cells) were seeded in 12-well plates and subjected to VP16 (40 μM) and/or HDACis (containing TSA 1 μM and NAM 5 mM) for 24 h. Then, cells were harvested, washed with PBS for twice times, following resuspended in binding buffer. According to cell apoptosis detection kit’s instructions (AF2020, LABLEAD, Beijing, China), cells were sequentially stained with 5 µL Annexin V-FITC (LABLEAD, Beijing, China) for 10 min. Subsequently, the cells incubated with 10 µL Prodium Iodide (PI, LABLEAD, Beijing, China) for 5 min at room temperature in the dark before analysis by flow cytometry.

### 4.5. Compare Antitumor Effect of Combination and Doxorubicin

To compare the antitumor efficacy of the VP16/TSA/NAM combination therapy with a first-line drug doxorubicin (Dox; D8740-25, Solarbio, Beijing, China), CCK-8 assays, morphological assessments and cell proliferation assays were performed as above experiments. A high dose of Dox (2 μM) was applied in these experiments according to the previous study [45]. Moreover, U2OS and SJSA-1 cells were exposure to these drugs for 48 h.

### 4.6. Western Blot

The experiment was conducted in accordance with previously established protocols [46]. Following drug treatment, proteins from U2OS or SJSA-1 cell were extracted with NP40 buffer containing 1% protease inhibitor cocktail (C0101, LABLEAD, Beijing, China) for 30 min on ice. Equal amounts of protein were combined with loading buffer, boiling for 10 min to denatured, resolved via sodium dodecyl sulfate-polyacrylamide gel electrophoresis, and subsequently transferred onto either 0.45 μm nitrocellulose membranes or 0.2 μm polyvinylidene fluoride membranes. Then, 5% skim milk were used to block membranes for at a minimum of 30 min at room temperature, and then incubation at 4 °C overnight with primary antibodies specific to target proteins, including Bcl-2 (sc-7382, Santa Cruz, Dallas, Texas, USA ), cleaved caspase-3 (9661, Cell Signaling Technology, Danvers, Massachusetts, USA), PARP (9542, Cell Signaling Technology, Danvers, Massachusetts, USA), YAP1 (13584-1-AP, Proteintech, Chicago, Illinois, USA), and beta-Actin (66009-1-Ig, Proteintech, Chicago, Illinois, USA). After incubation of primary antibody, membranes were incubated together with the secondary antibody labeled with horseradish peroxidase for 1 h behind washing three times at room temperature. Protein bands were visualized by a chemiluminescence detection system after incubation with Western ECL Substrate (1705060, Bio-Rad, Hercules, CA, USA). A densitometric analysis of the immunoreactive bands were exerted to quantify protein expression levels of post-treatment. The band intensities were measured by ImageJ software (Version 8.0_345 64-bit) and normalized against beta-actin.

### 4.7. RT-qPCR

After exposing U2OS and SJSA-1 cells to HDACis (TSA and NAM), VP16, and the combination of HDACis with VP16, as well as Dox or Dox/TSA/NAM for 24 h. Total RNA was subsequently extracted using TRIzol reagent (15596018CN, Invitrogen, Carlsbad, CA, USA) in accordance with the manufacturer’s protocol. cDNA was then synthesized employing the iScript™ reverse transcription supermix (1708841, Bio-Rad, Hercules, CA, USA). Quantitative RT-PCR was conducted with the iTaq™ universal SYBR^®^ green supermix (1725122, Bio-Rad, Hercules, CA, USA). Human β-Actin was used as the reference gene. The sequences of the specific PCR primers applied in this study are provide in Table 2.

### 4.8. RNA-Sequence Analysis

U2OS cells were exposed to HDACis (TSA and NAM), VP16, and the combination of HDACis with etoposide for a duration of 24 h. Total RNA was subsequently obtained by TRIzol reagent extraction. Following a serial of standard processes, cDNA libraries were constructed for high-throughput sequencing. The DESeq2 software (v1.34.0) conducted to analyze differential gene expression with Q value ≤ 0.05 (or FDR ≤ 0.001) after data filtering and variation detection. Gene expression differences among samples were visualized through heatmaps generated with the pheatmap package (version 1.0.12). The KEGG (https://www.kegg.jp/) enrichment analysis was performed using Phyper function of R software (v3.6.1) with Q value ≤ 0.05.

### 4.9. YAP Inhibitor Combination Experiment

To confirm the role of YAP1 in mediating the pro-apoptotic impact of the combination treatment, verteporfin (IV0350, Solarbio, Beijing, China) was employed as a YAP inhibitor. U2OS and SJSA-1 cells underwent various treatments, all lasting for 24 h: control group, VP16/TSA/NAM group, verteporfin (VP, 1 μM) group, and VP/VP16/TSA/NAM group. Following treatment, cell apoptosis proportions were performed to assess the role of the YAP inhibitor in combination with other therapeutic interventions.

### 4.10. Data Analysis

Experimental data are presented as mean ± SD. Difference among multiple groups were evaluated using either ordinary one-way ANOVA followed by Tukey’s multiple comparisons test or two-way ANOVA followed by Sidak’s multiple comparisons test. All statistical analyses were conducted utilizing GraphPad Prism software Version 8.0 (GraphPad Inc., San Diego, CA, USA). Significance levels of statistics are indicated as * *p* < 0.05, ** *p* < 0.01, and *** *p* < 0.001.

## 5. Conclusions

This research emphasizes the therapeutic efficacy of integrating VP16 with HDACis, in osteosarcoma cells. Our findings provide evidence that the HDACis improve the chemosensitivity of osteosarcoma cell lines to VP-16 by suppressing cellular proliferation and promoting apoptosis via caspase activation. Moreover, the combination therapy demonstrated a comparable effect on cell viability and exhibited a more significant inhibition of cell proliferation when compared to doxorubicin. Notably, this combination therapy significantly suppressed the Hippo/YAP signaling pathway, which is implicated in drug resistance in osteosarcoma, thereby improving the chemosensitivity to VP16.

## Figures and Tables

**Table 1:** The FA and CI values were calculated by CompuSyn after combination treatments of drugs in U2OS and SJSA-1 cells for 72 h.

VP16 (µM)	TSA (µM)	NAM (mM)	U2OS		SJSA-1		O-ASCs	
			FA	CI	FA	CI	FA	CI
2.5	0.0625	0.625	0.273	5.009	0.271	1.132	0.152	0.100
5	0.125	1.25	0.389	0.388	0.401	0.853	0.164	0.143
10	0.25	2.5	0.408	0.629	0.595	0.495	0.190	0.141
20	0.5	5	0.661	0.386	0.749	0.349	0.190	0.281
40	1	10	0.813	0.390	0.873	0.224	0.488	0.013

**Table 2:** A list of specific PCR primer sequences.

Gene Name	Nucleotide Sequence (5′-3′)
*BIRC5*	AGGACCACCGCATCTCTACAT
	AAGTCTGGCTCGTTCTCAGTG
*DLG5*	TGAGGCGATCCACCATGAG
	CCTCCCTGTATTTCTCCGACT
*PPP2R2A*	CATACCAGGTGCATGAATACCTC
	GGGTTATGTCTCGCTTTGTGTTT
*TEAD2*	CTTCGTGGAACCGCCAGAT
	GGAGGCCACCCTTTTTCTCA
*TGFB2*	CCATCCCGCCCACTTTCTAC
	AGCTCAATCCGTTGTTCAGGC
*YAP1*	TAGCCCTGCGTAGCCAGTTA
	TCATGCTTAGTCCACTGTCTGT
*GAPDH*	GACACCCACTCCTCCACCTTT
	TTGCTGTAGCCAAATTCGTTGT

## Data Availability

The novel data generated in this study have been made publicly accessible. These datasets are available in the NCBI SRA database (accession numbers: PRJNA1253891).

## Supplementary Materials

The following supporting information can be downloaded at: https://www.mdpi.com/article/10.3390/ijms26188935/s1.

## Abbreviations

ATCC	American Type Culture Collection
DEGs	Differential expression genes
DMEM	Dulbecco’s modified Eagle’s medium
Dox	Doxorubicin
FBS	Fetal bovine serum
GSEA	Gene Set Enrichment Analysis
HDACis	Histone deacetylases inhibitors
LATS1/2	Large tumor suppressor 1/2
MST1/2	Mammalian ste20-like kinases 1/2
NAM	Nicotinamide
O-ASCs	Omental adipose-derived stromal cells
PI	Prodium iodide
SAHA	Suberoylanilide hydroxamic acid
TAZ	Transcriptional coactivator with a PDZ-binding motif
TSA	Trichostatin A
VP16	Etoposide
YAP1	Yes-associated protein 1
