# Supplementary material for: HDAC Inhibitors Enhance the Chemosensitivity of Osteosarcoma Cells to Etoposide by Suppressing the Hippo/YAP Signaling Pathway
Source: Int J Mol Sci. 2025 Sep 13;26(18):8935. doi: 10.3390/ijms26188935 (PMC12469630; doi:10.3390/ijms26188935)
Supplement: Supplementary file 1 [file ijms-26-08935-s001.zip › ijms-3833871-supplementary.pdf]

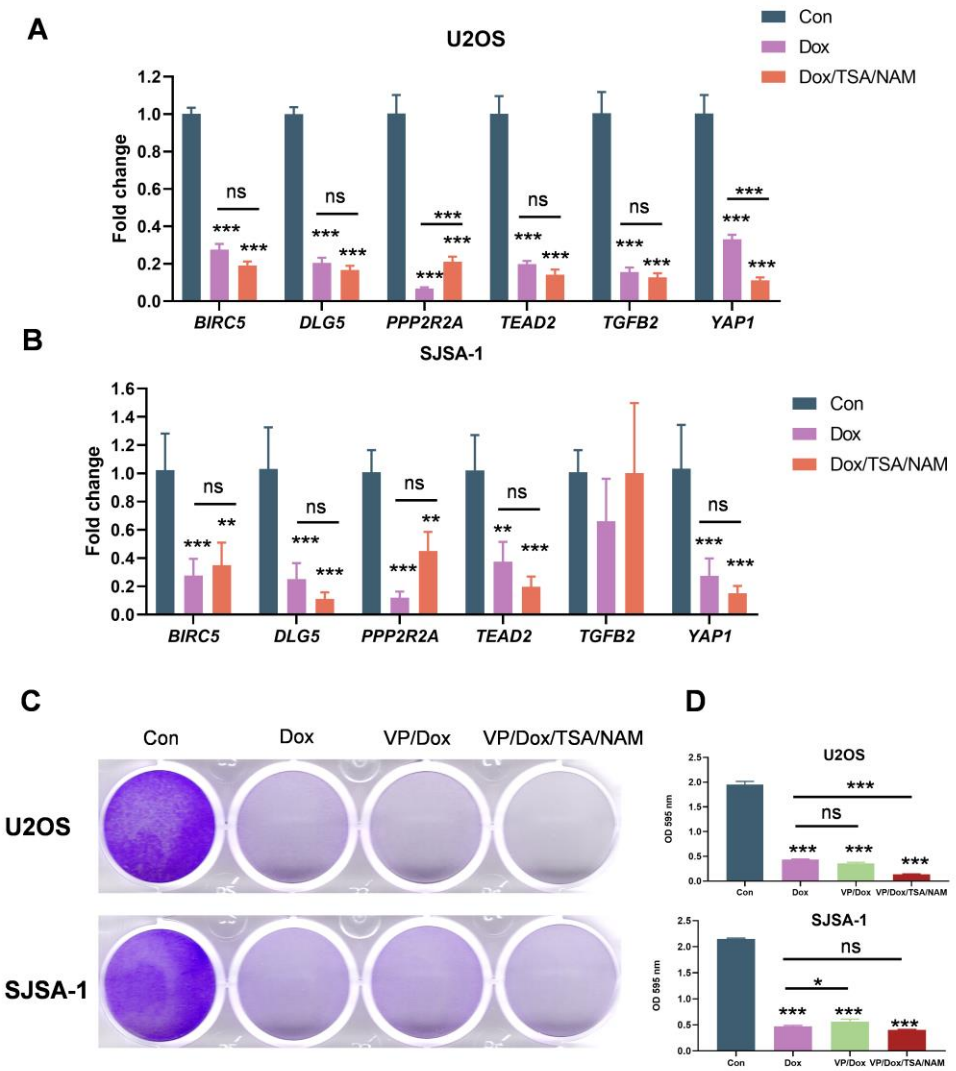

Figure S**1.** The Hippo signaling pathway was not implicated in the potential mechanisms associated with osteosarcoma cells treated with the combination of Dox/TSA/NAM. (A-B) RT-qPCR results of U2OS and SJSA-1 cells following 24 h treatments with either Dox (2 μM) or Dox/TSA/NAM (Dox 2 μM, TSA 1 μM and NAM 5 mM) treatments. (C-D) Cell proliferation analysis of U2OS and SJSA-1 cells treated with Dox alone, VP/Dox, Dox/TSA/NAM or VP/ Dox/TSA/NAM treatments (Dox 2 μM, VP 1 μM, TSA 1 μM, NAM 5 mM, VP16 40 μM) after 48 h. Data are presented as means ± SD from three replicates. *P* values were calculated using one-way ANOVA followed by Tukey's multiple comparisons test, and indicated by * $p < 0.05$, ** $p < 0.01$, *** $p < 0.001$ and ns (not significant).
